# Toward “English” Phonetics: Variability in the Pre-consonantal Voicing Effect Across English Dialects and Speakers

**DOI:** 10.3389/frai.2020.00038

**Published:** 2020-05-29

**Authors:** James Tanner, Morgan Sonderegger, Jane Stuart-Smith, Josef Fruehwald

**Affiliations:** ^1^Department of Linguistics, McGill University, Montreal, QC, Canada; ^2^Glasgow University Laboratory of Phonetics, University of Glasgow, Glasgow, United Kingdom; ^3^Department of Linguistics, University of Kentucky, Kentucky, KY, United States

**Keywords:** voicing effect, English, phonetic variability, Bayesian modeling, dialectal variation, speaker variability

## Abstract

Recent advances in access to spoken-language corpora and development of speech processing tools have made possible the performance of “large-scale” phonetic and sociolinguistic research. This study illustrates the usefulness of such a large-scale approach—using data from multiple corpora across a range of English dialects, collected, and analyzed with the SPADE project—to examine how the pre-consonantal Voicing Effect (longer vowels before voiced than voiceless obstruents, in e.g., *bead* vs. *beat*) is realized in spontaneous speech, and varies across dialects and individual speakers. Compared with previous reports of controlled laboratory speech, the Voicing Effect was found to be substantially smaller in spontaneous speech, but still influenced by the expected range of phonetic factors. Dialects of English differed substantially from each other in the size of the Voicing Effect, whilst individual speakers varied little relative to their particular dialect. This study demonstrates the value of large-scale phonetic research as a means of developing our understanding of the structure of speech variability, and illustrates how large-scale studies, such as those carried out within SPADE, can be applied to other questions in phonetic and sociolinguistic research.

## 1. Introduction

There exist a large number of well-studied properties of speech that are known to vary across languages and communities of speakers, which have long been of interest to sociolinguists and phoneticians. One dimension of this variability, which is the focus of this study, is that of variation *within languages*: across dialects and their speakers. For example, the deletion of word-final /t/ and /d/ segments (in e.g., *mist, missed*) has been shown to vary across a wide range of dialects and speech communities (e.g., Labov et al., 1968; Guy, 1980; Tagliamonte and Temple, 2005), as have the dialect-specific realization of English vowels (e.g., Thomas, 2001; Clopper et al., 2005; Labov et al., 2006), and variation in the degree of aspiration in English voiced and voiceless stops (e.g., Docherty, 1992; Stuart-Smith et al., 2015; Sonderegger et al., 2017). The study of this kind of variation provides a means of understanding the sources and structures of variability within languages: both in how particular dialects may systematically differ from each other, and how the variable realization of speech sounds maps to speakers' cognitive representation of language and speech (Liberman et al., 1967; Lisker, 1985; Kleinschmidt, 2018). Despite decades of research, however, there is much we do not know about the scope, extent, and structure of this kind of language-internal variability. Within the phonetic literature, most research has focused on highly-controlled speech styles in ‘laboratory settings', generally focusing on a single dialect in each study; much of the work focusing on phonetic variability in spontaneous speech is on single dialects (e.g., Ernestus et al., 2015). The sociolinguistic and dialectological literatures have often examined spontaneous speech, with some notable cross-dialectal studies (e.g., Clopper et al., 2005; Labov et al., 2006; Jacewicz and Fox, 2013), but nonetheless primarily focus on variation in vowel quality. Increasingly, however, research within phonetics and sociophonetics is being performed at a larger scale *across* speech communities (Labov et al., 2006, 2013; Yuan et al., 2006, 2007; Yuan and Liberman, 2014; Coleman et al., 2016; Liberman, 2018), driven by the development of new speech processing tools and data sharing agreements. This “large-scale” approach is applied here to one such well-studied variable, the pre-consonantal voicing effect, as a means of characterizing its degree and structure of variability in a single phonetic effect across English dialects and speakers.

The pre-consonantal voicing effect (henceforth *Voicing Effect*, VE) refers to vowels preceding voiced obstruents being consistently longer than their voiceless counterparts, such as the differences in *beat*-*bead* and *mace*-*maze* (House and Fairbanks, 1953; House, 1961). The VE has been reported—to greater or lesser extent—in a range of languages (Zimmerman and Sapon, 1958; Chen, 1970), though varies in size based on properties of the phonetic environment, such as whether the obstruent is a stop or fricative, the height of the vowel, and many others (Klatt, 1973; Crystal and House, 1982; Port and Dalby, 1982). The evidence for the English VE to date is sourced predominantly from laboratory studies of highly-controlled speech, often in citation form, recorded from small numbers of often standard General American English speakers (e.g., Rositzke, 1939; House and Fairbanks, 1953; Peterson and Lehiste, 1960; House, 1961; Crystal and House, 1982; Luce and Charles-Luce, 1985). On the basis of this evidence, the VE has been noted for being particularly large in English relative to other languages (Zimmerman and Sapon, 1958; Chen, 1970), and has long been suggested as a prominent cue to consonant voicing in English (Denes, 1955; Klatt, 1973). This in turn has motivated claims that the VE is learned in English, as opposed to being a low-level phonetic property in other languages (Fromkin, 1977; Keating, 2006; Solé, 2007). At the same time, numerous questions about the nature and extent of the VE in English remain unexplored. In this study, we will examine the variability in the VE across a range of English dialects, focusing on the following two research questions: (1) *how large is the VE as realized in spontaneous English speech?* and (2) *how much does the VE vary across dialects and speakers?* In addressing these questions, we hope to gain insight into a number of open issues, including the extent to which there is a single “English” VE or whether dialects differ in the magnitude of the effect, as well as the range of VE sizes across individual speakers of a given dialect.

This paper answers these questions by taking a “large-scale” approach to the study of the VE. Concretely, this refers to the use of a large amount of acoustic data, collected from a large number of speakers across a range of English dialects. This analysis falls within the framework of the *SPeech Across Dialects of English* (SPADE) project (Sonderegger et al., 2019, https://spade.glasgow.ac.uk/), which aims to consider phonetic and phonological variation in British and North American English across time and space through the use of automated acoustic analysis of features across English dialects occurring in many corpora. The methodological and research goals of the SPADE project are exemplified through this study of the English VE, specifically by the use of multiple corpora of diverse sources and structures, and the use of linguistic and acoustic analysis via the *Integrated Speech Corpus ANalysis* (ISCAN) tool (McAuliffe et al., 2019), developed as part of the broader SPADE project. Both the volume and complexity of the resulting data and the goals of the study motivate the need for appropriately-flexible approaches to the statistical analysis: specifically, the data is statistically analyzed using Bayesian regression models (Carpenter et al., 2017), which enable us to accurately estimate the size of the VE across dialects and speakers directly, whilst controlling for the complex nature of the spontaneous speech data.

The structure of this paper is as follows. Section 2 outlines previous work on the VE, and some of the outstanding questions related to our current understanding of its variability. Section 3 describes the data: the corpora of different dialects from SPADE. Sections 4, 5 describe the methodological approach: the process of acoustic and statistical analysis of the data. The results of this analysis are reported in section 6, and then discussed with respect to our specific research questions in section 7 and concluding in section 8.

## 2. The Voicing Effect (VE)

The observation that vowels preceding voiced obstruents are consistently longer than before voiceless obstruents was first noted in early phonetics textbooks (e.g., Sweet, 1880; Kenyon, 1940; Thomas, 1947; Jones, 1948) and in preliminary experimental work from the first half of the twentieth century (Heffner, 1937; Rositzke, 1939; Hibbitt, 1948). Studies explicitly manipulating the VE in English observed an effect of around 1.45—that is, vowels before voiced consonants were longer than before voiceless consonants by a ratio of around 2:3 (House and Fairbanks, 1953; House, 1961), and this effect was a cue to the voicing of the obstruent (Denes, 1955; Lisker, 1957; Raphael, 1972).

In these studies, VE was shown to be affected by consonant manner: namely, that fricatives showed a smaller or minimal VE compared to stops (Peterson and Lehiste, 1960), and less-robustly cued the voicing of the final consonant (Raphael, 1972). Initial studies of connected speech suggested that the size of the VE in this type of speech is more variable: VEs in carrier sentences are similar to those in isolated words (Luce and Charles-Luce, 1985)^1^ whilst vowels in read or spontaneous speech exhibit smaller VE sizes of around 1.2, and a negligible VE for fricatives (Crystal and House, 1982; Tauberer and Evanini, 2009). VE size is also modulated by the overall length of the vowel, which is hypothesized to be due to an intrinsic incompressibility of the vowel, limited by the minimal time required to perform the articulatory motor commands necessary for vowel production (Klatt, 1976). This general suggestion has been supported by observations that VE is smaller for unstressed and phrase-medial vowels (Umeda, 1975; Klatt, 1976), and vowels produced at a faster speech rate (Crystal and House, 1982; Cuartero, 2002). The VE is thus modulated by a range of phonetic factors, and largely predict a reduction of VE size in instances where vowels are generally shorter; vowels that undergo “temporal compression” have a reduced capacity to maintain a large VE size, and so VE is minimized. As these effects have only been investigated in laboratory speech, it is not clear whether the size and direction of these effects are maintained in less-controlled spontaneous speech styles.

Examining the VE across languages, Zimmerman and Sapon (1958) first observed that whilst English speakers produced a robust VE, Spanish speakers did not modulate vowel length in the same way, though this study did not control for the syllabic structure of test items. Comparing across English, French, Russian, and Korean, Chen (1970) observed that all four languages produced a VE size of at least 1.1, though all languages had different VE sizes (English = 1.63, French = 1.15, Russian = 1.22, Korean = 1.31). This was interpreted as evidence that VE is a phonetically-driven effect with additional language-specific phonological specification (Fromkin, 1977). Mack (1982), comparing English and French monolinguals with bilinguals, observed that English monolinguals maintained a substantially larger VE than French monolinguals, whilst the French-English bilinguals also produced the shorter French-style pattern instead of adapting to the larger English VE pattern. Keating (1985) suggested that VE is “phonetically-preferred,” though ultimately controlled by the grammar of the particular language. English, then, is expected to have a larger VE than other languages, though it is not known if the English VE is of a comparable size in spontaneous speech.

The work discussed above has not differentiated between varieties of English, and cross-linguistic comparisons of VE have presumed that a single “English” VE size exists. Little work has focused on variation in VE across English dialects beyond a small number of studies on specific dialects. One dialect group of interest has been Scottish Englishes and the application of the Scottish Vowel Length Rule (SVLR), where vowels preceding voiced fricatives and morpheme boundaries are lengthened, whilst all other contexts have short vowels (Aitken, 1981), and hence do not show the VE. In studies of the SVLR, some East Coast Scotland speakers show some evidence of the VE in production (Hewlett et al., 1999), whilst VE-like patterns were not observed in spontaneous Glaswegian (Rathcke and Stuart-Smith, 2016). On the other hand, studies of African American English (AAE) have claimed that voiced stops undergo categorical devoicing in this variety, which has resulted in additional vowel lengthing before voiced stops to maintain the pre-consonantal voicing contrast (Holt et al., 2016; Farrington, 2018). Only one study has previously compared the VE *across* English dialects in spontaneous speech. Tauberer and Evanini (2009), using interview data from the *Atlas of North American English* (Labov et al., 2006), observe that North American English dialects vary in their VE values, ranging from 1.02 to 1.33, and that dialects with shorter vowels on average (New York City) also show a smaller-than-average VE size (1.13). Moreover, despite recognition that individual speakers may exhibit variability in their VE sizes (Rositzke, 1939; Summers, 1987), no study has formally examined the extent of variability across speakers, nor how dialects may differ in the degree of VE variability amongst its speakers. The two patterns observed for Scottish and African American English suggest that English dialects can maintain relatively “small” (or no), and “large” VEs, respectively; we know little about the degree of VE variability beyond these dialects without a controlled study across multiple English varieties, which is one of the goals of this study.

Whilst a large number of studies on the VE have provided useful information for its realization in English and other languages, there are still a range of outstanding questions that can be addressed through a large-scale cross-dialectal approach. To what extent is the VE a *learned* property of a given language, compared with an *automatic* consequence of low-level phonetic structure? Much of the discussion with respect to variation in VE has revolved around differences across *languages* (Chen, 1970; Keating, 1985), which may differ both in their phonetic realization of segments but also the phonological representation of those segments. In this sense, examining VE variability internal to a language (i.e., across *dialects*) potentially avoids this problem; the specification of phonological categories—here, the voicing status of final obstruents—are expected be largely consistent within a language, meaning that language-internal variability may be driven by only differences in phonetic implementation.

Little is known about how English dialects may vary in their implementation of the VE, and so a range of possibilities exist for how dialects might compare. One possibility is that, with the exception of varieties with specific phonological rules interacting with the VE, dialects might cluster around a single “English” VE value, potentially of the size reported in the previous literature. Such a finding would support the previous approach in the literature, in terms of English compared to other languages, and suggest that dialects do not differ in how the final voicing contrast is phonetically implemented. Alternatively, dialects may differ gradiently from each other, and so may show a continuum of possible dialect-specific VE sizes. If dialects do differ in their VE size in this way, this would suggest that the previous literature on the VE in “English” accounts for just a fraction of the possible VE realizations across English, and would provide evidence that individual English dialects differ in their phonetic implementation of an otherwise “phonological” contrast (Keating, 1984, 1985).

Similarly, little is known about how individual speakers vary in the VE, and what the overall distribution of speaker VE sizes is. Synchronic variability across speakers is one of the key inputs to sound change (Ohala, 1989; Baker et al., 2011), and also defines the limits of a speech community, i.e., speakers who share sociolinguistic norms in terms of production and social evaluation (e.g., Labov, 1972). Whilst dialects may differ in the realization of segments or the application of phonological processes, dialect-internal variability is potentially more limited if a phonetic alternation such as the VE is critical to speech community membership.

## 3. Data for This Study

The varieties of English included in this study are from North America, Great Britain, and Ireland. For the purposes of this study, North American dialects refer to the regions of the United States and Canada outlined in *The Atlas of North American English*, which is based around phonetic, not lexical, differences between geographic regions (Labov et al., 2006; Boberg, 2018). For Canadian data specifically, the primary distinction was made between “urban” and “rural” speakers, based on its relative importance noted in comparison to much weaker geographic distinctions, at least for the corpus which makes up most Canadian data in this study (Rosen and Skriver, 2015). Within the British and Irish groups, dialects from England in this study are defined in terms of Trudgill's dialectal groupings (Trudgill, 1999), which groups regions in terms of both phonological and lexical similarity. Due to the lack of geographical metadata for speakers from Ireland and Wales, these dialects were simply coded as “Ireland” and “Wales” directly. Scottish Englishes are grouped based on information from *The Scottish National Dictionary*^2^. The data used in this study comes from the SPADE project, which aims to bring together and analyze over 40 speech corpora covering English speech across North America, the United Kingdom, and Ireland. In this study, we analyze data from 15 of these corpora, which together cover 30 different English dialects from these regions, comprised of speech from interviews, conversations, and reading passages. A basic description of each of these corpora is given below, outlining the type of speech and phonetic alignment tools used.

- *Audio British National Corpus* (AudioBNC, Coleman et al., 2012): The spoken sections of the British National Corpus, originally containing speech from over 1,000 speakers. However, due to a range of recording issues (e.g., overlapping speech, background noise, microphone interference), a large portion of the corpus is inaccurately aligned. In order to define a subset of the AudioBNC which maximizes the accuracy of the alignment, utterances were kept if they met a number of criteria: the utterance length was greater than one second, that the utterance contained at least two words, that the mean harmonics-to-noise ratio of the recording was at least 5.6, and that the mean difference in segmental boundaries between the alignment and a re-alignment with the Montreal Forced Aligner (MFA, McAuliffe et al., 2017a) was at most 30 ms^3^. 50 TextGrids from the remaining data were manually checked and deemed to be as approximately accurate as that of normal forced-alignment.
- *Brains in Dialogue* (Solanki, 2017): recordings of 24 female Glaswegian speakers producing spontaneous speech in a laboratory setting. There are 12 recordings for each speaker, which were aligned with LaBB-CAT (Fromont and Hay, 2012).
- *Buckeye* (Pitt et al., 2007): spontaneous interview speech of 40 speakers from Columbus Ohio, recorded in 1990s–2000s. The Buckeye corpus is hand-corrected with phonetic transcription labels: these were converted back to phonological transcriptions in order to be comparable with data from the other corpora.
- *Corpus of Regional African American Language* (CORAAL, Kendall and Farrington, 2018): spontaneous sociolinguistic interviews with 100 AAE speakers from Washington DC, Rochester NY, and Princeville NC, recorded between 1968 and 2016, and aligned with the MFA.
- *Doubletalk* (Geng et al., 2013): recordings of paired speakers carrying out a variety of tasks in order to elicit a range of styles/registers in a discourse/interactive situation. Ten speakers make up five pairs where one member is a speaker of Southern Standard British English and the other member is a speaker of Scottish English.
- *Hastings* (Holmes-Elliott, 2015): recordings of sociolinguistic interviews with 46 speakers from Hastings in the south east of England, male and female, aged from 8 to 90, aligned using FAVE (Rosenfelder et al., 2014).
- *International Corpus of English—Canada* (ICE-Canada, Greenbaum and Nelson, 1996): interview and broadcast speech of Canadian English, recorded in the 1990s across Canada, and aligned using the MFA. Speaker dialect was defined in terms of their city or town of origin. In this study, we coded a speaker as “urban” if their birthplace was a large Canadian city.
- *Canadian Prairies* (Rosen and Skriver, 2015): Spontaneous sociolinguistic interviews, recorded between 2010 and 2016, with speakers of varying ethnic backgrounds from the provinces of Alberta and Manitoba, conducted as part of the Language in the Prairies project, and was aligned using the MFA.
- *Modern RP* (Fabricius, 2000): reading passages by Cambridge University students recorded in 1990s and 2000s. The speakers were chosen for having upper middle-class backgrounds as defined by at least one parent having a professional occupation along with the speaker also having attended private schooling. The data used in this study come from a reading passage aligned with FAVE.
- *Philadelphia Neighborhood Corpus* (PNC, Labov and Rosenfelder, 2011): sociolinguistic interviews with 419 speakers from Philadelphia, recorded between 1973 and 2013, and were aligned with FAVE.
- *Raleigh* (Dodsworth and Kohn, 2012): semi-structured sociolinguistic interviews of 59 White English speakers in Raleigh, North Carolina, born between 1955 and 1989, and aligned with the MFA.
- *Santa Barbara* (Bois et al., 2000): spontaneous US English speech, recorded in the 1990s and 2000s, from a range of speakers of different regions, genders, ages, and social backgrounds.
- *The Scottish Corpus of Texts and Speech* (SCOTS, Anderson et al., 2007): approximately 1,300 written and spoken texts (23% spoken), ranging from informal conversations, interviews, etc. Most spoken texts were recorded since 2000.
- *Sounds of the City* (SOTC, Stuart-Smith et al., 2017): vernacular and standard Glaswegian from 142 speakers over 4 decades (1970s–2000s), collected from historical archives and sociolinguistic surveys, aligned using LaBB-CAT.
- *Switchboard* (Godfrey et al., 1992): 2,400 spontaneous telephone conversations between random participants from the multiple dialect regions in the United States on a variety of topics, containing data from around 500 speakers.

The goals of this study are to examine the size and variability in the English VE in spontaneous speech, and in variation in the VE across dialects and individual speakers. Specifically, the kind of dialectal variability being addressed in this study is that of *regional* variability: variability by race or ethnicity is not being directly considered in this study, with the exception of three African American English varieties, given the particular observations about AAE with respect to the VE (Holt et al., 2016; Farrington, 2018). This study also does not focus on differences according to age, either age-grading or apparent/real-time change in the VE over time; only speech data recorded since 1990s was included; the other data recorded prior to 1990 was excluded from further analysis. Analysis of the role of age and time in the VE in these English dialects remains a subject for future study.

## 4. Data Analysis

Having collected and organized the speech data into dialects, it is then possible to extract and acoustically analyze the data in the study: that is, going from raw data (audio and transcription files) to datasets which can be statistically analyzed. As the corpora differ in their formats—the phone labels used, organization of speaker data, etc.—modifying the acoustic analysis procedure for each different corpus format would be both labor and time-intensive, as well as increase the risk that the analysis itself differed across corpora. In order to standardize the acoustic analysis across corpora, the *Integrated Speech Corpus Analysis* (ISCAN) tool was developed for use in this kind of cross-dialectal study in the context of the SPADE project. This section provides a brief overview of the ISCAN system: see McAuliffe et al. (2017b, 2019) and the ISCAN documentation page for details of the implementation^4^.

The process of deriving a dataset from raw corpus files consists of three major steps. In the first step, individual speech corpora (in the form of sets of audio-transcription pairs) are *imported* into a graph database format, where each transcription file is minimally composed of word and phone boundaries (e.g., word-level and phone-level tiers in a TextGrid), and these word-phone relationships are structurally-defined in the database (i.e., that each phone belongs to a word). Importers have been developed for a range of standard automatic aligners, including all formats of corpora described in section 3. Corpora, represented in database format, can then be further *enriched* with additional structure, measurements, and linguistic information. For example, utterances can be defined as groups of words (separated silence of a specified length, e.g., 150 ms), syllables can be defined as a property between groups of adjacent phones. Once the database has been enriched with utterance and syllable information, speech rate (often defined as syllables per second within an utterance) can be calculated and included in the database. Similarly, information about words (such as frequency) or speakers (such as gender, age, dialect etc.) can be added to the corpus from metadata files. Once a corpus has been sufficiently enriched with linguistic and acoustic information, it is then possible to perform a *query* on the corpus at a given level of analysis. This level of analysis refers to the level of the hierarchy on which the resulting datafile should use as the main level of observation, for example individual phones, syllables, or utterances. Filters can be applied to a query to restrict it to the particular contexts of interest, for example, including only syllables occurring at the right edge of an utterance, or vowels followed by a specific subset of phone types (e.g., obstruents). Finally, the resulting query can then be *exported* into a data format (currently CSV only) for further analysis.

Each corpus was processed using the ISCAN software pipeline, and then combined into a single “master” dataset, containing all phonetic, dialect, and speaker information from all of the analyzed corpora necessary to carry out the analysis of the VE below. As the vowel duration annotations from the corpora (except for Buckeye) were created via forced alignment with a minimum duration of 10 ms and a time-step of 30 ms, any token with a vowel duration below 50 ms was excluded from further study, as is common in acoustic studies of vowel formants to exclude heavily reduced vowels (Dodsworth, 2013; Fruehwald, 2013). To reduce the additional prosodic and stress effects on vowel duration, the study only included vowels from monosyllabic words occurring phrase-finally, where a phrase is defined as a chunk of speech separated by 150 ms of silence. Raw speech rate was calculated as syllables per second within a phrase, from which two separate speech rates were derived. First, a mean speech rate for each speaker was calculated, which reflects whether a speaker is a “fast” or “slow” speaker overall. From that mean speech rate, a local speech rate was calculated as the raw rate for the utterance subtracted from the given speaker's mean. This local speech rate can be interpreted as how fast or slow that speaker produced the vowel within that particular phrase *relative* to their average speech rate (Sonderegger et al., 2017; Cohen Priva and Gleason, 2018). Word frequency was defined using the SUBTLEX-US dataset (Brysbaert and New, 2009). The final dataset contained 229,406 vowel tokens (1,485 word types) from 1,964 speakers from 30 English dialects. Table 1 shows the number of speakers and tokens for each dialect, and how many speakers/tokens were derived from each speech corpus.

**Table 1:** Number of speakers and tokens per dialect (left), and by corpora from which each dialect was derived.

**Region**	**Dialect**	***n* Speakers**	***n* tokens**	**Corpus**	***n* speakers**	***n* tokens**
North America	Canada (rural)	52	9,313	Canadian Prairies	44	8,316
				ICE-Canada	8	997
	Canada (urban)	64	12,124	Canadian Prairies	56	11,939
				ICE-Canada	8	185
	Midwest US	40	5,567	Buckeye	40	5,567
	New England	24	1,336	Santa Barbara	7	174
				Switchboard	17	1,162
	North Midland US	46	3,084	Switchboard	46	3,084
	Northern Cities US	21	1,377	Santa Barbara	21	1,377
	Northern US	58	3,086	Switchboard	58	3,086
	NYC	25	1,477	Santa Barbara	6	158
				Switchboard	19	1,319
	Philadelphia	371	59,581	PNC	371	59,581
	Princeville NC (AAE)	71	6,759	CORAAL	17	6,759
	Raleigh US	92	3,282	Raleigh	92	3,282
	Rochester NY (AAE)	14	6,308	CORAAL	14	6,308
	South Midland US	108	8,188	Switchboard	108	8,188
	Southern US	44	2,738	Santa Barbara	6	345
				Switchboard	38	2,393
	Washington DC (AAE)	50	21,205	CORAAL	50	21,205
	Western US	100	5,456	Santa Barbara	50	2,900
				Switchboard	50	2,556
United Kingdom & Ireland	Central Scotland	24	2,426	SCOTS	24	2,426
	East Central England	51	2544	Audio BNC	51	2,544
	East England	229	20,727	Audio BNC	132	6,622
				Doubletalk	5	726
				Hastings	44	12,642
				ModernRP	48	737
	Edinburgh	18	1,148	SCOTS	18	1148
	Glasgow	177	33,938	Brains in Dialogue	23	9,210
				SCOTS	27	2,294
				SOTC	127	2,2434
	Insular Scotland	8	351	SCOTS	8	351
	Ireland	19	624	Audio BNC	19	624
	Lower North England	60	3,325	Audio BNC	60	3,325
	North East England	17	488	Audio BNC	17	488
	Northern Scotland & Islands	33	2280	SCOTS	33	2,280
	Scotland	70	3,468	Audio BNC	65	2,633
				Doubletalk	5	835
	South West England	50	2,067	Audio BNC	50	2,067
	Wales	41	2,524	Audio BNC	41	2,524
	West Central England	41	2,615	Audio BNC	41	2,615
Total		1,964	229,406			

## 5. Statistical Analysis

The research goals of this study focus on the size and variability of the VE in English spontaneous speech, and how the VE varies across dialects and speakers. These goals motivate an approach of *estimating* the size of the VE in these contexts, rather than testing whether the VE “exists” or not. Whilst controlled laboratory experiments are explicitly designed to balance across these contexts (by including matching numbers of tokens with stops vs. fricatives, using words with similar frequency, etc.), spontaneous speech taken from corpora is rarely balanced in this sense: some speakers speak more than others, have different conversations leading to some combinations of segments occurring infrequently relative to others, speakers manage properties of their speech (such as speech rate) for communicative purposes which are generally absent in laboratory studies. In trying to obtain an accurate estimate of the VE (or indeed any other linguistic property), the unbalanced nature of spontaneous speech motivates the need for a statistical approach where individual factors of interest (e.g., obstruent manner of articulation, dialects, etc.) can be explored whilst controlling for the influence of other effects. This approach—the use of multiple regression to model corpus data—is now common in phonetics and sociolinguistic research (e.g., Tagliamonte and Baayen, 2012; Roettger et al., 2019), but has not, to our knowledge, been used to analyze multiple levels of variability in the VE.

In this study, this approach to estimation is performed using Bayesian regression modeling. Whilst other multifactorial statistical models would also be valid, Bayesian models provide us with some advantages that make the goal of estimating the size of the VE easier. Mixed-models are ideal for use in this study, as these capture variability at multiple levels (the VE overall, across dialects, across speakers) and this variability is of direct interest for our research questions. Bayesian mixed models resemble more traditional linear mixed-effects (LME) models approaches commonly used in linguistic and phonetic research, such as those performed with the *lme4* package (Bates et al., 2015), though differ in a few key respects. First, Bayesian models make it easy to calculate the *range* of possible VE sizes in each context, as opposed to a single value that would be output in LME models: whilst LME models provide ranges for “fixed” effects (across all dialects/speakers), Bayesian models provide a range of possible sizes for each level (i.e., an individual dialect). In a Bayesian model, all parameters (coefficients) in the model are assumed to have a *prior* distribution of possible values, reflecting which effect sizes are believed to be more or less likely, before examining the data itself. The output of a Bayesian model is a set of *posterior* distributions, which result from combining the priors and the likelihood of observing the data. Each model parameter has its own posterior distribution, which each represent the range of values for that parameter that is consistent with both the modeled data, conditioned on prior expectations about likely values, and the structure of the model itself. Bayesian models are well-suited to the task in this study, as they allow for flexible fitting of model parameters, and allow the complex random-effects structures which are often recommended for fitting statistically-conservative models (Barr et al., 2013), but which often fail to converge in LME models (Nicenboim and Vasishth, 2016). See Vasishth et al. (2018) for an introduction to Bayesian modeling applied to phonetic research.

A Bayesian mixed model of log-transformed vowel duration was fit using *brms* (Bürkner, 2018): a R-based front-end for the Stan programming language (Carpenter et al., 2017), containing the following population-level (“fixed effects”) predictors: the **voicing** and **manner** of the following obstruent, vowel **height** (high vs. non-high), the lexical **class** of the word (lexical vs. functional), both **mean** and **local** speech rates, and lexical **frequency**. To observe how compression of the vowel influences VE size, interactions between all of these factors with obstruent voicing were also included. The continuous predictors (both speech rates, frequency), were centered and divided by two standard deviations (Gelman and Hill, 2007). The two-level factors (obstruent voicing, manner, vowel height, lexical class) were converted into binary (0,1) values and then centered.

The group-level (“random effects”) structure of the model contained the complete set of model predictors for both dialects and speakers, nested within dialects. These terms capture two kinds of variability in the VE size: for each individual dialect, as well as the degree of variability across speakers—the nesting of speaker term inside dialects can be interpreted as capturing the variability in the size of the VE across speakers *within* a given dialect. Given the expectation that both the overall vowel duration (represented by the intercept) and the manner of the obstruent would affect the size of the VE, correlation terms between the intercept and both the consonant voicing and manner predictors, as well as for the interaction *between* the voicing and manner predictors, were included for both dialects and speakers. Random intercepts were included for words and phoneme labels, also nested within dialects. The model was fit using 8,000 samples across 4 Markov chains (2000/2000 warmup/sample split per chain) and was fit with weakly informative “regularizing” priors (Nicenboim and Vasishth, 2016; Vasishth et al., 2018): the intercept prior used a normal distribution with a mean of 0 and a standard deviation of 1 [written as *Normal*(0, 1)]; the other fixed effects parameters used *Normal*(0, 0.5) priors, with the exception of the obstruent voicing parameter which used a *Normal*(0.1, 0.2) prior^5^. The group-level (for dialects, speakers) parameters used the *brms* default prior of a half Student's *t*-distribution with 3 degrees of freedom and a scale parameter of 10. The correlations between group-level effects used the LKJ (Lewandowski et al., 2009) with ζ = 2, which gives lower prior probability to perfect (−1/1) correlations, as recommended by Vasishth et al. (2018).

## 6. Results

The results in this study will be reported in the context of the two main research questions concerning VE variability (1) in spontaneous speech, and (2) across English dialects and individual speakers. The results are reported for each effect in terms of the median value with 95% credible intervals (CrIs), and the probability of that effect's direction. These values enable us to understand the *size* of the effect (i.e., the change in vowel duration) and the confidence in the effect's predicted direction. The strength of evidence for an effect is distinct from the strength of the effect itself: to value the strength of evidence for an effect, we follow the recommendations of Nicenboim and Vasishth (2016) and consider there to be *strong* evidence of an effect if the 95% credible interval does not include 0, and *weak* evidence for an effect if 0 is within the 95% CrI but the probability of the effect's direction is at least 95% (i.e., that there is <5% probability that the effect changes direction). Evaluating the strength of an effect is determined with respect to effect sizes previously reported for laboratory (e.g., House and Fairbanks, 1953; House, 1961) and connected speech (Crystal and House, 1982; Tauberer and Evanini, 2009). The degree of variability across dialects can be compared with the findings of Tauberer and Evanini (2009); as there is no known comparison for speaker variability, this will be compared to variability across dialects as an initial benchmark.

### 6.1. The Voicing Effect in Spontaneous Speech

Table 2 reports the population-level (“fixed”) effects for each parameter in the fitted model. The “overall” VE size averaging across dialects, which is between 1.09 and 1.2, is estimated to be smaller than reported in previous laboratory studies ($\hat{\beta }$ = 0.14, CrI = [0.09, 0.19], Pr($\hat{\beta }>0$) = 1)^6^ and more consistent with VE sizes reported in studies of connected and spontaneous speech (Crystal and House, 1982; Tauberer and Evanini, 2009).

**Table 2:** Posterior mean ($\hat{\beta }$), estimated error, upper & lower credible intervals, and posterior probability of the direction of each population-level parameter included in the model of log-transformed vowel duration.

**Parameter**	**$\hat{\beta }$**	**Est.Error**	**95% CrI**	**Pr($\hat{\beta }$ < > 0)**
Intercept	−1.99	0.02	[−2.03, −1.96]	1
Obstruent voicing	0.14	0.03	[0.09, 0.19]	1
Obstruent manner	0.05	0.02	[0.02, 0.08]	1
Vowel height	−0.22	0.02	[−0.25, −0.18]	1
Lexical class	−0.14	0.03	[−0.21, −0.08]	1
Speech rate (mean)	−0.22	0.01	[−0.24, −0.20]	1
Speech rate (local)	−0.28	0.01	[−0.30, −0.26]	1
Lexical frequency	−0.05	0.01	[−0.08, −0.03]	1
Voicing : Manner	−0.04	0.03	[−0.10, 0.02]	0.91
Voicing : Height	0.07	0.02	[0.02, 0.11]	1
Voicing : Class	−0.07	0.03	[−0.13, 0.00]	0.97
Voicing : Mean rate	−0.01	0.01	[−0.03, 0.01]	0.77
Voicing : Local rate	−0.06	0.01	[−0.08, −0.03]	1
Voicing : Frequency	−0.07	0.02	[−0.11, −0.03]	1

**Figure 1:**
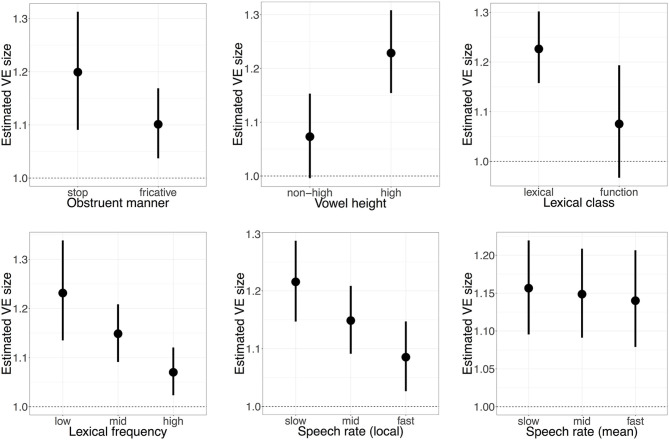
Modulation of VE size in different phonetic contexts: obstruent manner **(Top Left)**, vowel height **(Top Middle)**, lexical class **(Top Right)**, frequency **(Bottom Left)**, local **(Bottom Middle)**, and mean **(Bottom Right)** speech rates. Points and error bars indicate the posterior mean value with 95% credible intervals, whilst holding all other predictors at their average values. Dashed line indicates no difference between vowels preceding voiced or voiceless consonants. For continuous predictors (frequency, speech rates), the estimate VE size is shown at three values for clarity.

Looking at how the overall VE size for all dialects is modulated by phonetic context, there is weak evidence that the manner of the following obstruent modulates VE size ($\hat{\beta }$ = −0.04, CrI = [−0.10, 0.02], Pr($\hat{\beta }<0$) = 0.91): whilst stops appear to have a larger VE size (Figure 1, top left), the uncertainty in VE size for each obstruent manner (represented by the spread of the credible intervals) suggests that it is possible there is no difference in VE size between both obstruent manners. Whilst high vowels are shown to be shorter than non-high vowels overall ($\hat{\beta }$ = −0.22, CrI = [−0.25, −0.18], Pr($\hat{\beta }<0$) = 1), there is strong evidence that high vowels have a larger VE than non-high vowels ($\hat{\beta }$ = 0.07, CrI = [0.02, 0.11], Pr($\hat{\beta }>0$) = 1). There is a similarly strong effect for lexical class ($\hat{\beta }$ = −0.07, CrI = [−0.13, 0.00], Pr($\hat{\beta }<0$) = 0.97), where functional words have smaller VEs than open-class lexical items (Figure 1, top right). Lexical frequency also has a strong and evident effect on VE size ($\hat{\beta }$ = −0.07, CrI = [−0.11, −0.03], Pr($\hat{\beta }<0$) = 1), where higher-frequency words have smaller VEs than their lower-frequency counterparts (Figure 1, bottom left), whilst local speech rate also reduces VE size ($\hat{\beta }$ = −0.06, CrI = [−0.08, −0.03], Pr($\hat{\beta }<0$) = 1; Figure 1, bottom middle). For mean speaking rate, however, the effect on VE is both small with weak evidence ($\hat{\beta }$ = −0.01, CrI = [−0.03, 0.01], Pr($\hat{\beta }<0$) = 0.77): this is reflected in Figure 1 (bottom right), where the difference between faster and slower speakers has a negligible effect on VE size. These results generally suggest that shorter vowels (within-speaker) tend to have smaller VE sizes, consistent with the temporal compression account (Klatt, 1973): the apparent exception to this is the relationship between VE size and vowel height, which is addressed in section 7.

### 6.2. Voicing Effect Across Dialects and Speakers

Turning to dialectal variability in VE, we observe that the dialect variation in VE (the dialect-level standard deviation, ${\hat{\sigma }}_{dialect}$) is between 0.07 and 0.12: this can be interpreted as meaning that the difference in VE size between a “low” and “high” VE dialect is between 32 and 61%^7^ (Table 3). This is comparable with the range of possible values for the overall VE (between 0.09 and 0.19, Table 2, row 2). To understand whether this constitutes a “large” degree of variability, one metric is to assess whether a “low VE” dialect would actually have a reversed effect direction (voiceless > voiced), which is tested by subtracting 2 × ${\hat{\sigma }}_{dialect}$ from the overall VE size and comparing to 0. There is little evidence that dialects differ enough to change direction ($\hat{\beta }$ = −0.05, CrI = [−0.09, 0], Pr($\hat{\beta }>0$) = 0.06), which suggests that whilst individual dialects differ in the *size* of the VE, no dialect fully differs in the *direction* of the effect (i.e., no dialect's credible interval is fully negative).

**Table 3:** Posterior mean ($\hat{\sigma }$), estimated error, and 95% credible intervals for dialect and speaker-level parameters related to obstruent voicing included in the model of log-transformed vowel duration.

**Level**	**Parameter**	**$\hat{\sigma }$**	**Est.Error**	**95% CrI**
Dialect	Intercept	0.05	0.01	[0.03, 0.07]
	Obstruent Voicing	0.09	0.01	[0.07, 0.12]
	Voicing : Manner	0.12	0.02	[0.09, 0.16]
	Voicing : Height	0.04	0.01	[0.01, 0.06]
	Voicing : Class	0.06	0.01	[0.04, 0.09]
	Voicing : Mean Rate	0.02	0.01	[0.00, 0.05]
	Voicing : Local Rate	0.05	0.01	[0.03, 0.07]
Speaker	Intercept	0.10	0.00	[0.09, 0.10]
	Obstruent Voicing	0.08	0.00	[0.07, 0.08]
	Voicing : Height	0.11	0.01	[0.10, 0.12]
	Voicing : Manner	0.11	0.01	[0.10 0.13]
	Voicing : Class	0.13	0.01	[0.11, 0.14]
	Voicing : Local Rate	0.09	0.01	[0.08, 0.11]

**Figure 2:**
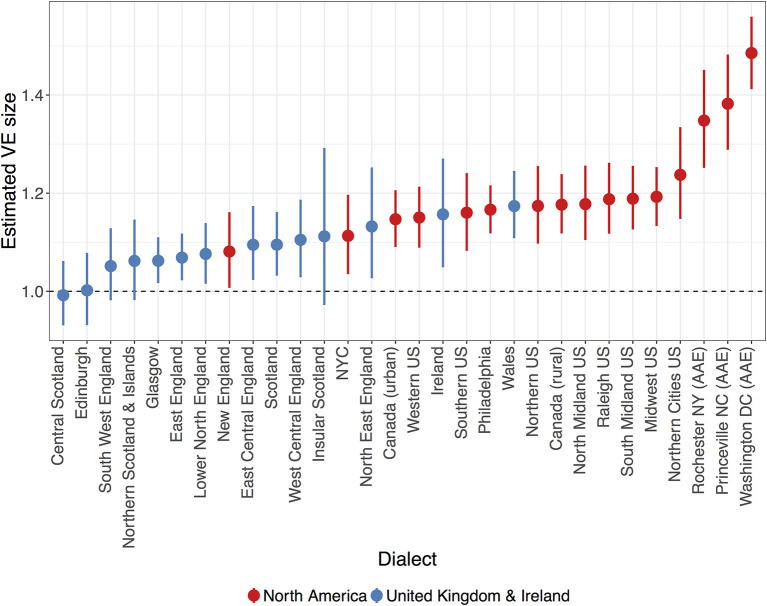
Estimated VE size for each dialect analyzed in this study (red = North American, blue = United Kingdom and Ireland). Points and errorbars indicate the posterior mean value with 95% credible intervals, whilst holding all other predictors at their average values. Dashed line indicates no difference between vowels preceding voiced or voiceless consonants.

Another way of understanding the degree of dialectal variability in VE is to examine the predicted VE for individual dialects. As shown in Figure 2, dialects appear to differ gradiently from each other, ranging from dialects with effectively-null VE to those with strong evidence for large VEs. The Scottish dialects of Central Scotland and Edinburgh have VEs of at most 1.06 and 1.09, respectively, based on their upper credible interval value, whilst their median values (indicated by the points in Figure 2) indicate that the most likely VE size is around 0 (Central Scotland: $\hat{\beta }$ = 0.99, CrI = [0.93, 1.06]; Edinburgh: $\hat{\beta }$ = 1.01, CrI = [0.93, 1.09]): indeed, all Scottish dialects have a predicted VE size of 1.16 at the highest, with most of these having median values <1.1 (Table 4). North American dialects, in contrast, all have robustly positive VE values (no credible interval crosses the 0 line) and are generally larger than the British and Irish variants, shown by the position of red (North American) and blue (United Kingdom and Ireland) points respectively in Figure 2. In particular, the AAE dialects have the largest VEs in the sample, which are all robustly larger than the average “English” VE size (Rochester NY: $\hat{\beta }$ = 1.35, CrI = [1.27, 1.44]; Princeville NC: $\hat{\beta }$ = 1.39, CrI = [1.31, 1.48]; Washington DC: $\hat{\beta }$ = 1.49, CrI = [1.42, 1.56]): this is consistent with previous studies of studies on AAE, which posit that final devoicing of word-final voiced obstruents results in compensatory vowel lengthening (Holt et al., 2016; Farrington, 2018).

**Table 4:** Estimated VE sizes (mean, estimated error, and upper and lower credible intervals) for each dialect used in this study.

**Dialect**	**$\hat{\beta }$**	**Est.Error**	**95% CrI**
Central Scotland	0.99	0.03	[0.93, 1.06]
Edinburgh	1.01	0.04	[0.93, 1.09]
South West England	1.05	0.03	[0.99, 1.12]
Glasgow	1.06	0.02	[1.02, 1.11]
Northern Scotland & Islands	1.06	0.04	[0.99, 1.14]
East England	1.07	0.02	[1.02, 1.12]
Insular Scotland	1.08	0.06	[0.96, 1.21]
Lower North England	1.08	0.03	[1.02, 1.15]
New England	1.08	0.04	[1.00, 1.17]
East Central England	1.09	0.03	[1.03, 1.16]
Scotland	1.10	0.03	[1.04, 1.16]
West Central England	1.11	0.03	[1.04, 1.18]
NYC	1.12	0.04	[1.04, 1.20]
North East England	1.14	0.05	[1.04, 1.26]
Canada (urban)	1.15	0.02	[1.09, 1.21]
Western US	1.15	0.03	[1.09, 1.21]
Canada (rural)	1.17	0.03	[1.12, 1.24]
Ireland	1.17	0.04	[1.07, 1.28]
Philadelphia	1.17	0.02	[1.12, 1.22]
Southern US	1.17	0.03	[1.10, 1.24]
North Midland US	1.18	0.03	[1.11, 1.26]
Northern US	1.18	0.03	[1.11, 1.26]
Wales	1.18	0.03	[1.11, 1.25]
Raleigh US	1.19	0.03	[1.13, 1.26]
South Midland US	1.19	0.03	[1.13, 1.26]
Midwest US	1.20	0.03	[1.14, 1.26]
Northern Cities US	1.24	0.04	[1.15, 1.33]
Rochester NY (AAE)	1.35	0.03	[1.27, 1.44]
Princeville NC (AAE)	1.39	0.03	[1.31, 1.48]
Washington DC (AAE)	1.49	0.02	[1.42, 1.56]

**Figure 3:**
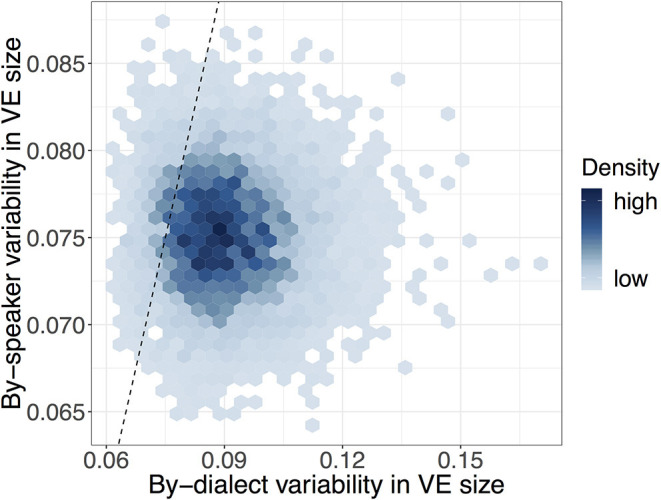
Heatmap of posterior samples of by-dialect (${\hat{\sigma }}_{dialect}$) and by-speaker (${\hat{\sigma }}_{speaker}$) voicing effect standard deviations. Equal variability is indicated by the dashed line, with darker shades indicating a greater density of samples.

Turning to variability in VE across individual speakers, we observe that speakers are estimated to vary within-dialect by between 0.07 and 0.08 (${\hat{\sigma }}_{speaker}$ = 0.08, CrI = [0.07, 0.08]), meaning that speakers differ in their VE ratios by between 32 and 37% (Table 3). To put this value in context and get an impression of the size of variability across speakers, this value is compared with the degree of variability across dialects. Figure 3 illustrates how likely the model deems different degrees of by-speaker and by-dialect variability: highest probability (darker shading) lies where by-dialect variability is greater than by-speaker variability. By the metric of between-dialect variability, Figure 3 illustrates that whilst dialects differ in VE size, individual speakers vary little from their dialect-specific baseline value.

## 7. Discussion

The findings from this study will be discussed with respect to the two research questions: (1) how the VE is realized in spontaneous speech, and (2) how the VE varies across dialects and speakers. The VE in English is often considered to be substantially larger than in other languages (Chen, 1970) and claimed to play a significant perceptual role in cueing consonant voicing (Denes, 1955). Taken together, these observations have formed the basis for claims that the VE in English is phonologically specified beyond an otherwise phonetically-consistent acoustic property across languages (Fromkin, 1977; Keating, 1985). Previous work has focused on controlled laboratory speech, leaving open the question of how the VE is realized in spontaneous English speech.

In this study, the overall VE in spontaneous speech was observed to have a maximum size of around 1.2—substantially smaller than the 1.5 commonly reported in laboratory studies (e.g., House and Fairbanks, 1953; Peterson and Lehiste, 1960; House, 1961; Chen, 1970), and more consistent with previous research on VE in connected speech (Crystal and House, 1982; Tauberer and Evanini, 2009). Spontaneous VE size was also shown to be affected by a range of phonetic factors, such as consonant manner, vowel height, frequency, and speech rate, though the evidence for each of these effects varies substantially (section 6.1). What the effects of these phonetic factors suggest is that contexts where vowels are often shorter also have shorter VE sizes, supporting the argument of “temporal compression”: that vowels which have already undergone shortening cannot be subsequently shortened further (Harris and Umeda, 1974; Klatt, 1976). An interesting exception to this finding is that the VE size was found to be larger for high vowels than non-high vowels in this study (Figure 1)—the direction of this effect may be counter to that predicted by temporal compression, and opens a question as to whether this and other predictions of temporal compression are straightforwardly replicable in spontaneous speech environments. The overall smaller-size and impact of phonetic factors of the VE in spontaneous speech indicates a possible fragility of the VE in spontaneous speech, in apparent contrast to the supposed perceptual importance of the VE as a cue to consonant voicing (Denes, 1955; Lisker, 1957; Raphael, 1972). This apparent conflict between the perceptual importance of the VE and its subtlety in production provides an interesting area for future work.

The fact that VE size in English differs so widely between laboratory and connected speech not only demonstrates the importance of speech style and context on phonetic realization (Labov, 1972; Lindblom, 1990), but also raises the question of “how big” the VE in English really is, or could be. If larger overall VE size is only observable in laboratory speech, it would be interesting to empirically re-evaluate the question of whether English VE is in fact larger than in other languages. For languages that exhibit smaller VEs than English in laboratory speech (Chen, 1970), it is not clear how such languages may realize the VE in more naturalistic speech. One possibility is that the VE across languages is comparatively small in spontaneous speech and similarly affected by phonetic factors; alternatively, the VE in spontaneous speech across other languages may still be smaller than in English and retain cross-linguistic differences akin to those reported by Chen (1970), and thus English would still retain its status as a language with a distinct realization of the VE.

The first research question (section 6.1) considered how the VE was modulated in spontaneous speech, averaging across dialects. To what extent dialects themselves differ in VE was the focus of the second research question. As shown in section 6.2, English was shown to exhibit a range of different VE sizes across individual dialects. The dialects with the smallest and largest VEs—Scottish Englishes and AAE, respectively—were expected to show these values given evidence of additional phonological rules governing vowel duration in these varieties (Aitken, 1981; Holt et al., 2016; Rathcke and Stuart-Smith, 2016; Farrington, 2018). Beyond these varieties, dialects appear to differ gradiently from each other, ranging in VE values from around 1.05 in South West England to 1.24 in the Northern Cities region (Figure 2). As opposed there being a single “English” VE value, there appears to be a range of VE sizes within the language. Such a finding further complicates the notion that English has a particular and large VE relative to other languages. Imagining these different dialects as “languages” with minimally different phonological structures, this finding demonstrates that such similar “languages” can have very different phonetic effects (Keating, 1985). This in turn underlies a more nuanced approach to the question of whether English truly differs from other languages in its VE size: not only may English have varieties with greater or lesser VE sizes, but other languages may also exhibit similar dialectal VE ranges.

Individual speakers are also shown to vary in the realization of the VE, though the extent of this variability is rather limited when compared to variability across dialects (Figure 3): that is, whilst dialects appear to demonstrate a range of possible VE patterns, individual speakers vary little from their dialect-specific baseline values. Such a finding supports an interpretation where the VE has a dialect-specific value which speakers learn as part of becoming a speaker of that speech community. The limited extent of speaker variability could predict that the VE will be stable within individual English dialects, given the key role of synchronic speaker variability as the basis for sound change (Ohala, 1989; Baker et al., 2011). This would need checking on a dialect-by-dialect basis, however, given recent evidence of Glaswegian undergoing weakening in its vowel duration patterns (Rathcke and Stuart-Smith, 2016). It also highlights the need for studies addressing both synchronic and diachronic variability across dialects, which we hope to address in future work. One important caveat to the finding is that it assumes that all the dialects analyzed in this study contain only speakers who are speakers of that dialect: if a given dialect had a particularly large degree of by-speaker variability, it could be that this could reflect the existence of multiple speakers of different dialects (and thus different VE patterns) within that particular dialect coding. This is unlikely to be a particular problem in this study, however, as a separate model that allows for by-speaker variability to vary on a per-dialect basis showed that no dialect with a sufficiently large number of tokens exhibited overly large by-speaker variability (section 6.2).

By using speech data from multiple sources and multiple dialects, it has been possible to investigate variability of a phonological feature across “English” overall, examine variability at the level of individual dialects and speakers, and reveal the extent of English-wide phonetic variability that was not previously apparent in studies of individual dialects and communities. In this sense, our “large-scale” approach, using consistent measures and controlling factors, enables us to understand the nature of dialectal variability in the English VE directly within the context of both other dialects and English as a whole.

Whilst this kind of study extends the scope of analysis for (socio)phonetic research, there are of course a number of limitations that should be kept in mind in studies of this kind. This study of the English VE predominantly uses data from automatic acoustic measurements, in turn calculated from forced aligned-segmented datasets. All forced-alignment tools have a minimum time resolution (often 10 ms), a minimum segment duration (often 30 ms), and there always exists the possibility of poor or inaccurate alignment. This is a necessary consequence of the volume of data used in this study: there is simply *too much* data to manually check and correct all durations, and so the best means of limiting these effects is through sensible filtering and modeling of the data. For example, segments with aligned durations of less than 50 ms were excluded, since accurately capturing the duration of a vowel this small could be difficult given the time resolution of the aligner. This decision could exaggerate the size of the VE estimation, as only the most reduced vowels have been removed from the data. Another property of forced alignment which impacts our study of VE is that aligners will only apply the phonological segment label to the segment, meaning that it is possible to only examine VE in terms of *phonological* voicing specification (i.e., whether a segment is underlyingly voiced or not), as opposed to whether the segment itself was realized with phonetic voicing. For example, the realization of the stop as devoiced (Farrington, 2018) or as a glottal stop (Smith and Holmes-Elliott, 2018), or the relative duration of the closure preceding the vowel (Lehiste, 1970; Port and Dalby, 1982; Coretta, 2019), could affect VE size which is not controllable by exclusively using phonological segment labels. How this kind of phonetic variation, and the more general relationship between a “phonological” and a “phonetic” VE, should be understood would certainly be an interesting project for future work. Finally, given the diversity of formats and structures of the corpora available for this study, it has only been possible to categorize and study dialects in a rather broad “regional” fashion. Similarly, we were unable to investigate the effect of speaker age due to the heterogenous coding of age across the corpora: we agree this is an important dimension that we have attempted to account for in the approach to statistical modeling, and is certainly necessary to examine in future work. Whilst these limitations may be less suitable for approaching other questions in phonetics and sociolinguistics which are concerned with variability at a more detailed level, the approach taken in this study points to a promising first step toward exposing the structures underlying fine-grained phonetic variability at a larger level across multiple speakers and dialects of a language.

## 8. Conclusion

The recent increase in availability of spoken-language corpora, and development of speech and data processing tools have now made it easier to perform phonetic research at a “large-scale”—incorporating data from multiple different corpora, dialects, and speakers. This study applies this large-scale approach to investigate how the English Voicing Effect (VE) is realized in spontaneous speech, and the extent of its variability across individual dialects and speakers. Little has been known about how the VE varies across dialects bar a handful of studies of specific dialects (Aitken, 1981; Tauberer and Evanini, 2009; Holt et al., 2016). English provides an interesting opportunity to directly examine how phonetic implementation may differ across language varieties with minimally different phonological structures (Keating, 1985). By applying tools for automatic acoustic analysis (McAuliffe et al., 2019) and statistical modeling (Carpenter et al., 2017), it was found that the English VE is substantially smaller in spontaneous speech, as compared with controlled laboratory speech, and is modulated by a range of phonetic factors. English dialects demonstrate a wide degree of variability in VE size beyond that expected from specific dialect patterns such as the SVLR, whilst individual speakers are relatively uniform with respect to their dialect-specific baseline values. In this way, this study provides an example of how large-scale studies can provide new insights into the structure of phonetic variability of English and language more generally.

## Data Availability Statement

The datasets generated for this study are available on request to the corresponding author.

## Author Contributions

JT extracted the data, performed the statistical analysis, and wrote the first draft of the manuscript. All authors contributed to the conception and design of the study. All authors contributed to manuscript revision, and read and approved the submitted version.

## Conflict of Interest

The authors declare that the research was conducted in the absence of any commercial or financial relationships that could be construed as a potential conflict of interest.

## Footnotes

^1^Harris and Umeda (1974), in their study of overall vowel duration, attribute this difference to a “mechanical” prosody as a consequence of numerous repetitions.

^2^Part of *The Dictionary of the Scots Language* (https://dsl.ac.uk/).

^3^We are grateful to Michael Goodale for designing and performing this filtering protocol.

^4^https://iscan.readthedocs.io/

^5^The values chosen for the obstruent voicing parameter reflect the decision to allow a wide range of possible VE sizes, including values both above and below those reported in the previous literature. A sensitivity analysis was performed using an additional model fit with a “uniform” flat prior for the obstruent voicing parameter, which returned VE values differing by an order of 10^−3^, suggesting that the decision for the weakly-informative prior did not adversely affect the reported results.

^6^As vowel duration was log-transformed prior to fitting, effects are interpreted by taking the exponent of the model parameter's value, e.g., *e*^0.19^=1.2, which refers to a vowel duration increase of 20%.

^7^The value is multiplied by 4 to get the 95% range of values = $2{\hat{\sigma }}_{dialect}$ for both sides of the distribution = 0.28, which is then back-transformed from log via the exponential function = *e*^0.28^ = 1.32.
